# Supplementary material for: The Role of Extracellular Matrix Expression, ERK1/2 Signaling and Cell Cohesiveness for Cartilage Yield from iPSCs
Source: Int J Mol Sci. 2019 Sep 2;20(17):4295. doi: 10.3390/ijms20174295 (PMC6747490; doi:10.3390/ijms20174295)
Supplement: Supplementary file 1 [file ijms-20-04295-s001.pdf]

Supplementary Materials

Table S1. Mean microarray expression levels of top significantly higher expressed genes in iMPCs compared to MSCs.

Gene Name	Accession Number	Mean iMPC	Mean MSC	Fold Difference
LOC646723	XR_017241.1	3092	149	20.8
MMP1	NM_002421.2	2185	117	18.7
TUBB2B	NM_178012.3	2135	138	15.5
SPP1	NM_000582.2	1002	99	10.1
PTPRF	NM_002840.3	2068	206	10.1
CD24	NM_013230.2	854	90	9.4
RGS4	NM_005613.3	3671	393	9.3
KRT8	NM_002273.2	821	90	9.1
TFAP2C	NM_003222.3	737	84	8.8
TMSB15A	NM_021992.2	2096	245	8.6
PRAGMIN	NM_001080826.1	2068	252	8.2
ZIC2	NM_007129.2	696	87	8.0
ITGA2	NM_002203.3	1209	162	7.5
COL4A5	NM_000495.3	607	87	7.0
LOC644743	XR_016703.1	628	90	7.0
FLJ40504	NM_173624.1	657	95	6.9
SLC2A1	NM_006516.1	1647	242	6.8
SHISA2	NM_001007538.1	755	112	6.8
F2RL1	NM_005242.3	870	140	6.2
MSX1	NM_002448.3	3015	511	5.9
CSRP2	NM_001321.1	1105	192	5.8
ANKRD1	NM_014391.2	593	105	5.7
C5orf46	NM_206966.2	571	101	5.6
NTSR1	NM_002531.2	521	93	5.6
ODZ3	NM_001080477.1	669	122	5.5
CLDN1	NM_021101.3	470	86	5.5
COL13A1	NM_080805.2	1639	308	5.3
SLC7A5	NM_003486.5	1469	285	5.2
FBLN2	NM_001004019.1	904	177	5.1
DKFZp761P0423	XM_291277.4	695	136	5.1
CPE	NM_001873.1	420	84	5.0
EDN1	NM_001955.2	491	100	4.9
DPPA4	NM_018189.3	391	80	4.9
AGPAT9	NM_032717.3	812	168	4.9
SPINT2	NM_021102.2	444	92	4.8
OXTR	NM_000916.3	690	149	4.6
DSG2	NM_001943.2	393	86	4.6

NOX4	NM_016931.2	391	88	4.4
KRT18P13	XM_001726959.1	394	90	4.4
FABP5L2	XM_001134012.2	1595	364	4.4
BEXL1	XM_936467.2	801	184	4.4
CDCA7	NM_031942.4	741	173	4.3
CDK6	NM_001259.5	1167	276	4.2
MAMDC2	NM_153267.3	387	92	4.2
NFE2L3	NM_004289.5	403	96	4.2
FABP5	NM_001444.1	740	180	4.1
IGFBP5	NM_000599.2	9595	2341	4.1
PCDHB2	NM_018936.2	483	121	4.0
EEF1A2	NM_001958.2	346	87	4.0
IL11	NM_000641.2	523	134	3.9
AURKB	NM_004217.2	685	178	3.9
BCAT1	NM_005504.4	1757	456	3.9
BEX4	NM_001080425.1	483	128	3.8
NCAPG	NM_022346.3	1073	289	3.7
VIL2	NM_003379.3	2890	778	3.7
DENND2A	NM_015689.2	458	124	3.7
LOC642956	XM_938166.3	597	164	3.6
CDC20	NM_001255.2	4054	1119	3.6
LOC100216001	NR_024475.1	322	89	3.6
CCDC99	NM_017785.3	738	207	3.6
MGC39900	NM_194324.1	822	232	3.5
UHRF1	NM_013282.3	1210	344	3.5
SEMA3A	NM_006080.2	526	151	3.5
BCAR3	NM_003567.2	1051	303	3.5
TUFT1	NM_020127.1	478	139	3.5
EZR	NM_003379.4	4597	1339	3.4
CCNA2	NM_001237.2	802	234	3.4
UCHL1	NM_004181.3	4259	1250	3.4
MARCKSL1	NM_023009.4	1917	565	3.4
SFRP1	NM_003012.3	4122	1215	3.4
PFKFB3	NM_004566.2	896	264	3.4
UBE2C	NM_181800.1	1118	332	3.4
RPL29	NM_000992.2	1067	318	3.4
HBEGF	NM_001945.1	601	179	3.4
MLLT11	NM_006818.3	3126	933	3.4
AHNAK2	NM_138420.2	484	145	3.3
LYPD1	NM_144586.5	1004	303	3.3
C11orf41	NM_012194.1	422	127	3.3
PSAT1	NM_021154.3	346	107	3.2

CENPF	NM_016343.3	838	260	3.2
LYN	NM_002350.1	315	98	3.2
CPVL	NM_031311.3	257	80	3.2
AFF3	NM_001025108.1	253	80	3.2
ZNF185	NM_007150.2	468	149	3.2
LIN28B	NM_001004317.2	251	81	3.1
VAT1L	NM_020927.1	303	99	3.1
C14orf78	XM_001132404.1	429	139	3.1
STMN1	NM_203399.1	491	160	3.1
LMNB2	NM_032737.2	1792	585	3.1
CCNB2	NM_004701.2	1561	511	3.1
SOX11	NM_003108.3	394	129	3.1
CDH2	NM_001792.2	3189	1046	3.1
SLC38A1	NM_030674.3	247	82	3.0
SPC24	NM_182513.1	937	312	3.0
NUAK1	NM_014840.2	510	171	3.0
DNAJC9	NM_015190.3	1727	579	3.0
FRMD4A	NM_018027.3	1044	350	3.0
LOC731049	XM_001129232.1	2080	700	3.0
LOC647954	XR_018676.1	263	89	3.0

Table S2. Mean microarray expression levels of top significantly lower expressed genes in iMPCs compared to MSCs.

Gene Name	Accession Number	Mean iMPC	Mean MSC	Fold
C10orf116	NM_006829.2	85	3654	-42.8
PENK	NM_006211.2	86	3578	-41.8
DCN	NM_001920.3	91	2168	-23.9
IL6	NM_000600.1	88	1791	-20.4
AKR1C3	NM_003739.4	168	3383	-20.1
CSRP1	NM_004078.1	97	1914	-19.7
VCAM1	NM_080682.1	90	1748	-19.4
RASD1	NM_016084.3	116	2075	-17.8
CD74	NM_001025159.1	85	1493	-17.5
ALPL	NM_000478.3	101	1676	-16.5
MEG3	NR_002766.1	90	1394	-15.6
EBF3	NM_001005463.1	96	1466	-15.2
TMEM119	NM_181724.1	80	1213	-15.2
ENPP2	NM_001040092.1	98	1473	-15.0
LAMA4	NM_002290.2	135	1914	-14.1
FMO3	NM_001002294.1	83	1126	-13.6
COX7A1	NM_001864.2	83	1040	-12.6
EPSTI1	NM_033255.2	104	1304	-12.6
IRX3	NM_024336.1	353	4187	-11.9
RAC2	NM_002872.3	152	1795	-11.9
CTHRC1	NM_138455.2	158	1865	-11.8
ALDH1A3	NM_000693.2	89	1027	-11.6
LOC730415	XM_001124749.2	88	983	-11.2
CRYAB	NM_001885.1	93	1037	-11.1
LBP	NM_004139.2	81	884	-11.0
TNFRSF14	NM_003820.2	94	1024	-10.9
AKR1C4	NM_001818.2	97	1051	-10.9
LUM	NM_002345.3	106	1131	-10.6
COL6A3	NM_004369.2	642	6729	-10.5
PDGFRA	NM_006206.3	266	2782	-10.5
CEBPD	NM_005195.3	142	1484	-10.4
ACTA2	NM_001613.1	603	6254	-10.4
EPDR1	NM_017549.3	223	2302	-10.3
ABI3BP	NM_015429.2	96	975	-10.1
HCP5	NM_006674.2	88	886	-10.0
SLC1A3	NM_004172.3	99	993	-10.0
CCL2	NM_002982.3	239	2326	-9.8
PPAP2B	NM_003713.3	264	2493	-9.5

VAMP5	NM_006634.2	200	1839	-9.2
APOE	NM_000041.2	120	1054	-8.8
KRTAP1-5	NM_031957.1	89	773	-8.7
ANPEP	NM_001150.1	146	1268	-8.7
LY96	NM_015364.2	130	1121	-8.6
PLAC9	NM_001012973.1	94	810	-8.6
FAP	NM_004460.2	105	889	-8.5
COL6A1	NM_001848.2	770	6490	-8.4
RARRES3	NM_004585.3	80	668	-8.3
RCN3	NM_020650.2	164	1348	-8.2
PDE7B	NM_018945.3	168	1361	-8.1
DLX5	NM_005221.5	79	633	-8.0
DHRS3	NM_004753.4	109	873	-8.0
KCTD12	NM_138444.2	91	717	-7.9
CA12	NM_001218.3	272	2108	-7.8
PTGES	NM_004878.3	148	1137	-7.7
LRRC32	NM_005512.1	131	993	-7.6
C7orf10	NM_024728.1	213	1588	-7.5
CTSK	NM_000396.2	116	858	-7.4
HMOX1	NM_002133.1	137	986	-7.2
XYLT1	NM_022166.3	86	608	-7.1
TIMP3	NM_000362.4	213	1505	-7.1
SULF1	NM_015170.1	140	969	-6.9
ENG	NM_000118.1	266	1824	-6.9
KIAA1644	XM_936510.2	88	602	-6.9
IFI27	NM_005532.3	84	571	-6.8
NFIX	NM_002501.2	274	1840	-6.7
FOXC1	NM_001453.1	615	4114	-6.7
TGFBR3	NM_003243.2	133	886	-6.7
IFI44L	NM_006820.1	96	634	-6.6
NNMT	NM_006169.2	366	2403	-6.6
OLFML3	NM_020190.2	154	999	-6.5
LTBR	NM_002342.1	90	571	-6.3
GAS6	NM_000820.1	364	2283	-6.3
ADAMTS1	NM_006988.3	294	1838	-6.3
PCOLCE	NM_002593.2	542	3379	-6.2
ADCY4	NM_139247.2	91	565	-6.2
IGFBP4	NM_001552.2	1063	6557	-6.2
RDH5	NM_002905.2	121	739	-6.1
C1S	NM_201442.1	86	527	-6.1
UBA7	NM_003335.2	89	541	-6.1
FLJ10916	NM_018271.2	84	511	-6.1

IFI44	NM_006417.3	114	687	-6.0
CAT	NM_001752.2	85	510	-6.0
TXNIP	NM_006472.2	606	3627	-6.0
COL16A1	NM_001856.3	164	978	-6.0
MGC87042	XM_001128032.1	322	1898	5.9
ARHGEF3	NM_019555.1	200	1173	5.9
LXN	NM_020169.2	504	2922	5.8
LOC730743	XM_001127014.1	85	490	5.8
TMEM173	NM_198282.1	109	625	5.7
GPX7	NM_015696.3	124	712	5.7
PNPO	NM_018129.2	177	1012	5.7
CRISPLD2	NM_031476.2	192	1084	5.7
CD248	NM_020404.2	611	3430	5.6
DBC1	NM_014618.2	84	468	5.5
FAM20A	NM_017565.2	86	472	5.5
IFI30	NM_006332.3	88	486	5.5
VASN	NM_138440.2	460	2516	5.5
PRRX2	NM_016307.3	442	2415	5.5
EBF1	NM_024007.2	104	547	5.2

Table S3. Mean microarray expression levels of typical endodermal markers in expanded iMPCs and MSCs.

Gene Symbol	iMPC	MSC	Fold Difference
AFP	122	b	1.4
CLDN8	b	b	1.0
CLIC6	b	b	1.0
CXCR4	b	b	1.0
FOXA1	b	b	1.0
FOXA2	b	b	1.0
GATA1	b	b	1.0
SOX17	b	b	1.0
SOX7	b	b	1.0
ST14	b	b	1.0
TMPRSS2	b	b	1.0
TMPRSS4	b	b	1.0

b – below detection level

Table S4. Mean microarray expression levels of typical ectodermal markers in expanded iMPCs and MSCs.

Gene Symbol	iMPC	MSC	Fold Difference
TUBB3	3843	3097	1.2
FABP7	b	b	1.0
FOXD3	b	b	1.0
GBX2	b	b	1.0
GFAP	b	b	1.0
HES5	b	b	1.0
MAP2	b	b	1.0
NEUROG2	b	b	1.0
OTX2	b	b	1.0
PAX6	b	b	1.0
PROM1	b	b	1.0

b – below detection level

Table S5. Mean microarray expression levels of genes classified to the integrin signaling pathway according to the PANTHER algorithm.

Gene Symbol	iMPC	MSC	Fold Difference
LAMA4	135	1914	-14.1
RAC2	152	1795	-11.9
COL6A3	642	6729	-10.5
ACTA2	603	6254	-10.4
COL6A1	770	6490	-8.4
COL16A1	164	978	-6.0
COL6A2	186	969	-5.2
COL8A1	929	3854	-4.2
MAPK3	365	1385	-3.8
ITGBL1	b	264	-2.8
LAMB2	317	794	-2.5
ITGB5	805	1961	-2.4
LAMA3	b	196	-2.3
MAPK13	170	341	-2.0
ACTB	11110	8272	1.3
BCAR1	265	175	1.5
RAC3	148	b	1.6
ITGA6	164	b	2.0
RHOD	137	276	2.0
PTK2	1326	527	2.5
COL13A1	1639	308	5.3
COL4A5	607	b	7.0

b – below detection level

Table S6. Primers used for qPCR.

Gene	Forward (5′→3′)	Reverse (5′→3′)	Annealing Temp.
RPL13	CATTTCTGGCAATTTCTACAG	CAGGCAACGCATGAGGAAT	58°C
HPRT	AAGGGTGTTTATTCCTCATGGA	CCTCCCATCTCCTTCATCAC	58°C
CPSF6	AAGATTGCCTTCATGGAATTGAG	TCGTGATCTACTATGGTCCCTCTCT	58°C
COL6A3	CACAACAAAGCCTGTAACCACC	CTGTTATCTCAAACACCTGGAC	58°C
DCN	TCCCAACTTAGCCAAATTATTC	ACACAACTCTGCTAGACCTG	58°C
LAMA4	GCCAAGAACTGTGCAGTGTG	AGCTTATGGTTGGGCAGTCC	60°C
LUM	ATGCCACACCACAAGATCCC	ACCACCAATCAATGCCAGGA	58°C
